# Wide-Angle Viewing System versus Conventional Indirect Ophthalmoscopy for Scleral Buckling

**DOI:** 10.1038/srep13256

**Published:** 2015-09-02

**Authors:** Yohei Tomita, Toshihide Kurihara, Atsuro Uchida, Norihiro Nagai, Hajime Shinoda, Kazuo Tsubota, Yoko Ozawa

**Affiliations:** 1 Department of Ophthalmology, Keio University School of Medicine, Tokyo, Japan

## Abstract

Wide-angle viewing systems (WAVSs) were originally established for pars plana vitrectomy. However, their application to scleral buckling surgery was recently reported. In this study, we compared the outcomes of scleral buckling using a noncontact WAVS with that of scleral buckling using conventional indirect ophthalmoscopy for rhegmatogenous retinal detachment. The clinical records of 39 eyes (39 patients) with rhegmatogenous retinal detachment primarily treated between November 2012 and June 2014 at the Vitreo-Retina Surgical Division Clinic at the Department of Ophthalmology, Keio University Hospital were retrospectively reviewed. Scleral bucking was performed using WAVS with surgical placement of an endoilluminator in 16 eyes and indirect ophthalmoscopy in 23 eyes. The patients in these groups were consecutive over different intervals. The preoperative demographics, success rate of retinal reattachment, intraoperative findings, and postoperative complications were evaluated. There were no significant differences in pre- or postoperative conditions between groups, and similar surgical outcomes were achieved with the WAVS and conventional procedures. However, compared with the conventional procedure, the WAVS procedure resulted in fewer intraoperative corneal epithelial disorders (p = 0.049) and decreased the surgical duration of segmental buckling (p = 0.02); therefore, it may be suggested as an effective alternative procedure.

Although recent progress in pars plana vitrectomy (PPV) has expanded its application to rhegmatogenous retinal detachment, scleral buckling remains a valuable surgical procedure and is the first-line treatment for particular cases, such as those involving rhegmatogenous retinal detachment caused by retinal holes in the lattice in young patients. Although an attempt to improve the method of scleral buckling by the substitution of indirect ophthalmoscopy with a noncontact wide-angle viewing system (WAVS) was recently made^1^,^2^,^3^, no study has compared the surgical outcomes of the two procedures.

Scleral buckling is a well-established procedure for treating rhegmatogenous retinal detachment. However, conventional scleral buckling using indirect ophthalmoscopy has several limitations, which surgeons endeavor to resolve through further modifications. In the conventional procedure, extensive indentation for cryopexy may cause inflammation; moreover, the surgical field cannot be shared during surgery or recorded on video, thus compromising the support provided by surgical assistants and co-medical staff as well as resident education. The aforementioned issues were mostly resolved through the use of a noncontact WAVS during surgery, which requires lesser indentation for observing the breaks and effects of cryopexy. Furthermore, during indentation, the surgical field can be visualized through the microscope and/or can be recorded, making it convenient for patients, surgeons, assistants, and residents in need of training^1^,^2^,^3^.

Although the advantages of WAVS-assisted scleral buckling are well accepted, an objective comparison of its outcomes with those of conventional scleral buckling using indirect ophthalmoscopy has not been performed. Therefore, in this study, we compared the two procedures with regard to the success rate of retinal reattachment, intraoperative findings, and postoperative complications in order to elucidate the effectiveness of WAVS-assisted scleral buckling and establish its use.

## Results

### Patient demographics

In this study, we included 23 eyes of 23 patients (18 men and 5 women; average age, 45.3 ± 12.5 years; range, 23–68 years) who underwent scleral buckling with conventional indirect ophthalmoscopy and 16 eyes of 16 patients (10 men and 6 women; average age, 41.3 ± 14.0 years; range, 24–65 years) who underwent scleral buckling with WAVS. The surgical procedure was selected depending on the period; the conventional procedure was selected in the initial period and WAVS was selected in the later period. All procedures other than the method of fundus visualization were identical in both groups, with the exception of the use of viscoelastic material in the WAVS group. As a standard, viscoelastic material is generally used with WAVS during PPV^4^. The average postoperative follow-up period was 10.4 months, ranging from 6 to 24 months.

There were no significant differences between the two groups with regard to patient age (p = 0.32), gender (p = 0.29), the localization of retinal breaks, the extent of retinal detachment (p = 0.35), the preoperative best-corrected visual acuity (BCVA; logMAR, p = 0.23), and the spherical equivalent refractive error (p = 0.05; Table1). Only one eye in the WAVS group contained an intraocular lens; all the other patients in both groups exhibited phakic eyes.[Table t1]

### Intra- and postoperative findings

No significant difference was observed in the rate of retinal reattachment after the first surgery between the WAVS (93.8%) and conventional (95.7%) groups (p = 0.79; Table 2). Furthermore, no significant differences were observed in the postoperative BCVA (p = 0.28) between groups, and the postoperative spherical equivalent refractive error was comparable with the preoperative error (p = 0.39). Although there was no significant difference in the overall surgical duration between the WAVS and conventional groups (p = 0.07), the surgical duration was significantly shorter in the WAVS group than in the conventional group when the analysis was limited to patients who underwent segmental buckling (p = 0.02).

An intraoperative complication, namely retinal herniation at the time of external subretinal fluid drainage occurred in one eye in the conventional group. A significant difference was observed in the number of eyes that underwent corneal epithelial peeling due to intraoperative epithelial edema between the WAVS (n = 0) and conventional groups (n = 5; p = 0.049).

There was a trend for a higher incidence of postoperative corneal epithelial disorders in the conventional group (n = 7) compared with that in the WAVS group (n = 1), although the difference was not significant (p = 0.07). Postoperative complications included one case of macular pucker in the WAVS group (p = 0.23) and one case each of cataract formation (p = 0.4), central serous chorioretinopathy (p = 0.4), and macular edema (p = 0.4) in the conventional group.

## Discussion

In this study, we compared the rate of retinal reattachment and postoperative BCVA between patients treated by scleral buckling using WAVS and those treated by scleral buckling using conventional indirect ophthalmoscopy. We found no significant differences between the two groups with regard to both parameters. In addition, we found that the intraoperative corneal condition was better and the surgical duration for segmental buckling was shorter in patients treated by WAVS-assisted scleral buckling. We found no surgical complications related to scleral incision and intraocular insertion of the endoilluminator in this study, although such a concern was theoretically raised.

To date, several studies have described the technique for scleral buckling using WAVS and its surgical outcomes. These reports recommend the use of WAVS in cases of undetected retinal breaks^1^ and show that WAVS generally has favorable surgical outcomes^2^,^3^,^5^. However, no study thus far has directly compared WAVS with the conventional method for scleral buckling, and to our knowledge, the present case series represents the first comparative study of these procedures based on clinical chart reviews.

The findings of this study showed that patients treated with noncontact WAVS had significantly better ocular surface conditions during surgery. This was probably because the retinal breaks could be easily observed with less scleral indentation because of the wide surgical view through the noncontact WAVS, thus inducing a smaller increase in the intraocular pressure and placing less stress on the cornea. We placed a viscoelastic material on the cornea when using the noncontact WAVS, which is commonplace during WAVS-assisted PPV^4^; this step may also have contributed to the maintenance of a better corneal condition. The viscoelastic material was not used in the conventional group, because it is not used in the conventional buckling procedure.

Contact lenses placed during PPV may also avoid corneal drying, but they have a narrow surgical view that can be slightly disadvantageous; however, contact lenses were not included for analysis in this study. Because noncontact WAVS provides a good surgical view in cases of corneal opacity and small pupils^4^, WAVS can prove advantageous for the treatment of rhegmatogenous retinal detachment accompanied by such complications.

**Figure 1:**
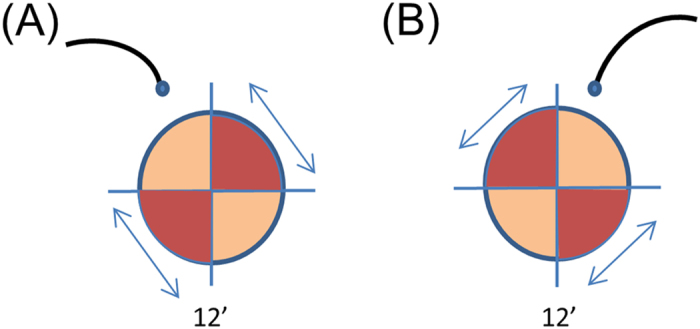
Settings of the endoilluminator. The endoilluminator is placed at the inferior part of the sclera and within the area where the conjunctiva has been prepared for suturing the buckle. The endoilluminator is placed at the 5 o’clock position when the retinal tears are identified between the 6 and 9 o’clock positions or between the 12 and 3 o’clock positions (A), while it is placed at the 7 o’clock position when the tears are identified between the 3 and 6 o’clock positions or between the 9 and 12 o’clock positions (B).

The shorter surgical duration with WAVS-assisted segmental buckling may be attributed to the wide surgical field, which allows the surgeon to mark the breaks easily for cryopexy. The surgical duration for WAVS-assisted encircling buckling showed a decreasing trend, although there were no significant differences, probably because the surgical duration included the time spent on procedures other than marking, cryopexy, and final check of the fundus. There was no limitation in scleral depression because a perilimbal conjunctival incision was made. We had examined the fundus carefully to identify the breaks before surgery; therefore, the cannula was appropriately placed according to the rule shown in Fig. 1 and did not interfere with scleral indentation. The retina near the incision point of the cannula was lightened by tilting the latter. At this time, we had to carefully avoid touching the lens. An endoilluminator with a smaller gauge, e.g., 27G, may be easier to light around the incision site.

**Figure 2:**
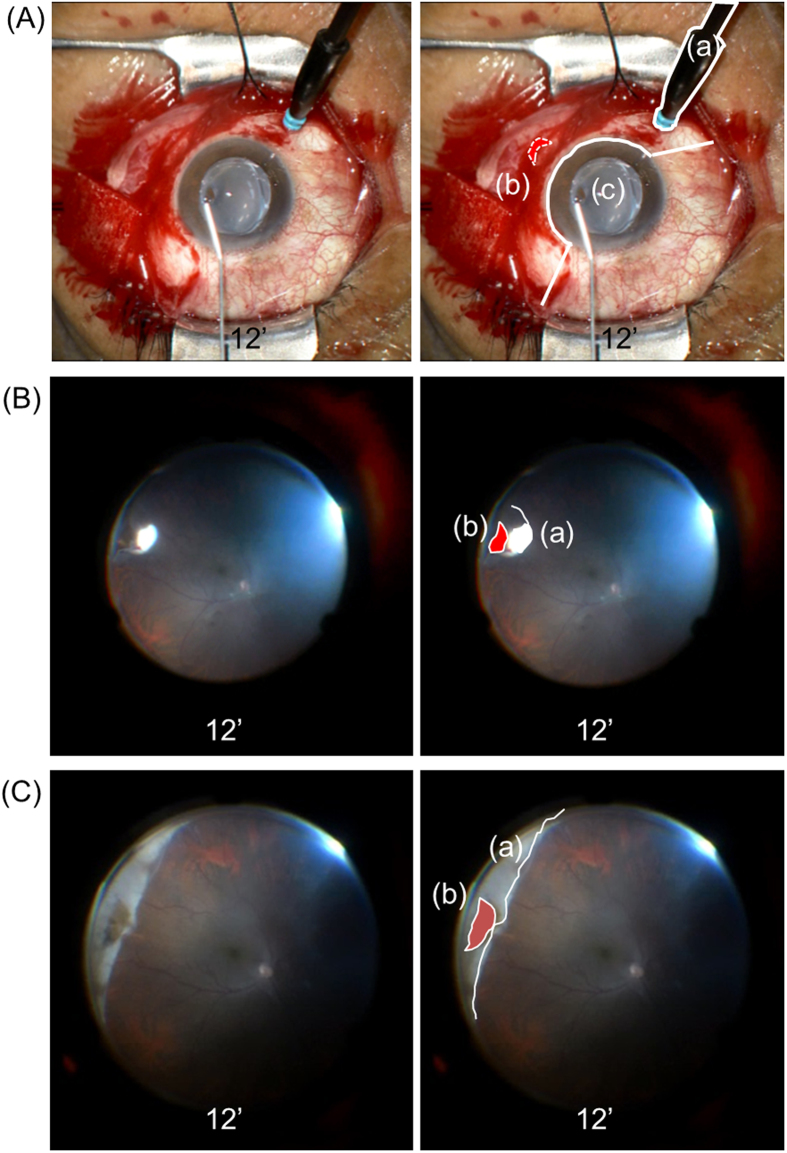
Intraoperative records for patients who underwent scleral buckling using a wide-angle viewing system (WAVS). Intraoperative surgical field: Right panels are overlaid with schemes. (**A**) An endoilluminator is placed at the 7 o’clock (**a**) position, because the retinal tear is at the 4 o’clock (**b**) position. A viscoelastic material (Artz Dispo^TM^) is placed on the cornea (**c**). (**B**) Cryopexy is performed at the posterior site (**a**) of the retinal tear at the 4 o’clock (**b**) position using WAVS. (**C**) The localization and height of the scleral buckle (**a**) are confirmed by both the surgeon and assistants using WAVS [(**b**) the retinal tear].

With WAVS, the surgical field can be reviewed in video recordings (Fig. 2) and shared in real-time with surgical assistants, co-medical staff, and medical students. Therefore, WAVS is advantageous in terms of not only the surgical output and educational benefits but also clinical studies such as the present one.

The scleral buckling procedure may sometimes injure the neck and back of surgeons because of the posture required to perform this procedure. The use of WAVS enables surgeons to sit down and maintain a comfortable posture during the entire procedure^6^. This aspect, however, was not objectively evaluated in the current study.

A few disadvantages of WAVS are the costs involved and facilities required. Trocar cannulas and endoilluminators required for WAVS are disposable instruments. Furthermore, in one patient (incidence, 6.3%) in the WAVS group, macular pucker developed as a postoperative complication and necessitated an additional surgery. Nevertheless, the incidence of this postoperative complication is similar to that reported with the conventional procedure (4%–8.5%)^7^,^8^,^9^.

This study has some limitations. The sample size was relatively small, while the follow-up period was relatively short. A study with a larger sample size or a prospective study is required to precisely compare the effects of these surgical methods in the future. Theoretical surgical complications related to scleral incision and intraocular insertion of the endoilluminator include vitreous wick from the scleral wound, endophthalmitis, lens damage, and light toxicity^1^. Although these complications did not occur in this study, a longer follow-up of a larger population should be performed before a conclusion regarding the use of WAVS is reached.

In the current study, the WAVS and conventional procedures showed similar rates of retinal reattachment, postoperative BCVA, and retinal complications. Moreover, intraoperative corneal epithelial disorders were less frequent. Furthermore, this procedure may be more conducive to resident training. Although further studies using a larger study group and longer follow-up period are required, WAVS may be considered an alternative standard procedure for scleral buckling in the future.

## Methods

This study was conducted according to the guidelines laid down in the Declaration of Helsinki, and all procedures involving human subjects were approved by the ethics committee at Keio University School of Medicine. Informed consent was obtained from all subjects.

### Subjects

The subjects included 39 patients (28 men and 11 women; average age, 43.6 ± 13.1 years; range, 23–68 years) diagnosed with rhegmatogenous retinal detachment and surgically treated primarily with scleral buckling alone by a single surgeon (YT) at the Vitreo-Retina Surgical Division of the Department of Ophthalmology, Keio University Hospital between November 2012 and June 2014. Patients requiring simultaneous cataract surgery and those who underwent retinal reattachment by PPV with or without scleral buckling were excluded. All the conventional scleral buckling procedures were performed for 23 eyes during the first 7 months of the study, i.e., from November 2012 to May 18, 2013, while all the WAV-assisted procedures were performed for 16 eyes during the next 14 months, i.e., from May 24, 2013 to June 2014.

### Eye examinations

All patients underwent preoperative evaluation using the Landolt C chart, slit-lamp examination, and binocular indirect ophthalmoscopy after pupil dilation with 0.5% tropicamide and 0.5% phenylephrine. The area of retinal detachment, number and localization of retinal break(s), and macular involvement were evaluated (Table 1). The rate of retinal reattachment after the first surgery and intra- and postoperative complications were recorded (Table 2).

### Surgical procedure

Local anesthesia was induced by the retrobulbar injection of 2% lidocaine. The sclera was exposed with a perilimbal conjunctival incision. Once the extraocular muscles were hooked at the insertion site, tractional sutures were placed with 4-0 black silk.

Then, during scleral buckling with the noncontact WAVS, a scleral incision was placed with a 25G trocar cannula at a distance of 4 mm from the corneal limbus for the insertion of an endoilluminator (Chandelier Lighting System, Alcon, Fort Worth, Texas, USA). The position of the sclerotomy was selected according to the localization of the break (Fig. 1). The sclerotomy was created in the same quadrant as the break to avoid additional invasion of the conjunctiva. A plug was inserted into the trocar when the endoilluminator was removed from the trocar without a check valve. A viscoelastic material (ARTZ Dispo^TM^, Seikagaku Kogyo Co, Tokyo, Japan) was placed on the corneal surface during surgery (Fig. 2A).

Fundus observation was performed through the noncontact WAVS (Resight; Carl Zeiss Meditec AG, Jena, Germany or BIOM; Oculus, Germany) using the endoilluminator. The localization of the retinal breaks was identified and cryopexy was performed through WAVS (Fig. 2B). After the endoilluminator was removed, a silicone sponge (#506 or #506G MIRA, USA) or a silicone tire (#277 MIRA, USA) with or without a silicone band and tube (#240 and #270 MIRA, USA) was fixed to the sclera with mattress sutures using 5-0 polyester (Alcon, Fort Worth, Texas, USA). Then, the endoilluminator was reinserted to confirm the adequacy of the position and height of the buckle (Fig. 2C). Subretinal fluid drainage was performed using an argon laser after slcerotomy and diathermy^10^,^11^,^12^. Finally, after checking the fundus through WAVS, the 25G cannula was removed, followed by cotton swab compression. The conjunctiva was closed with 8-0 silk sutures.

During conventional scleral buckling using indirect ophthalmoscopy, fundus observation was performed through an indirect ophthalmoscope without a cannula to mark the breaks and perform cryopexy. The silicone materials were placed and subretinal fluid was drained using the same methods used in the WAVS group. The position and height of the buckle were confirmed by indirect ophthalmoscopy. Physiological saline irrigation was employed during surgery to prevent the corneal surface from drying.

### Statistical analysis

All values are expressed as means ± standard deviations. The results were compared using the Mann–Whitney U test. All statistical analyses were performed using SPSS (version 22.0, SPSS Japan, Tokyo, Japan). A p-value of <0.05 was considered statistically significant.

## Additional Information

**How to cite this article**: Tomita, Y. *et al.* Wide-Angle Viewing System versus Conventional Indirect Ophthalmoscopy for Scleral Buckling. *Sci. Rep.* **5**, 13256; doi: 10.1038/srep13256 (2015).

## Figures and Tables

**Table 1 t1:** Preoperative demographics.

	Conventional	WAVS	P-value
Number of patients	23	16	
Age range (years)	23–68	24–65	
Mean ± SD	45.3 ± 12.5	41.3 ± 14.0	0.32
Gender (Men; %)	18 (78.3)	10 (62.5)	0.29
Follow-up periods (months)	6–24	6–15	
Mean ± SD	11.7 ± 6.8	8.6 ± 3.2	0.29
BCVA (logMAR)	0.11 ± 0.39	0.01 ± 0.24	0.23
Spherical equivalent refractive error	−3.61 ± 3.14	−5.81 ± 3.24	0.05
Number of retinal breaks per patient	1.4 ± 0.7	1.6 ± 0.6	0.22
Number of patients with holes (%)	9 (39.1)	7 (43.8)	0.77
Tears (%)	10 (43.5)	9 (56.3)	0.44
Dialyses (%)	4 (17.4)	0 (0)	0.08
Location of retinal breaks			
Limited to superior quadrants	16 (69.9)	8 (57.1)	0.22
Limited to inferior quadrants	5 (21.7)	4 (25)	0.81
Both superior and inferior quadrants	2 (8.7)	4 (25)	0.17
Extent of retinal detachment			
< 2 quadrants	8 (34.8)	8 (50)	0.35
≥2 quadrants	15 (65.2)	8 (50)	0.35
Macular detachment	4 (17.4)	2 (12.5)	0.68

WAVS, wide-angle viewing system; BCVA, best-corrected visual acuity

*P < 0.05.

**Table 2:** Intra- and postoperative findings.

	Conventional	WAVS	P-value
**Intraoperative findings**			
Buckling			
Segmental	18 (78.3)	12 (75)	0.81
360° circumferential	5 (21.7)	4 (25)	0.81
External subretinal fluid drainage	8 (34.8)	9 (56.3)	0.3
Intraoperative complications			
Retinal herniation	1 (4.3)	0	0.4
Corneal epithelial disorder	5 (21.7)	0	0.049*
Surgical duration (min)	130 ± 46	107 ± 41	0.07
Segmental buckling (min)	117 ± 34	92 ± 33	0.02*
**Postoperative findings**			
Retinal reattachment			
With single surgery	22 (95.7)	15 (93.8)	0.79
Final	23 (100)	16 (100)	1
Postoperative BCVA (LogMAR)	−0.01 ± 0.11	−0.04 ± 0.08	0.28
Spherical equivalent refractive error	−3.45 ± 3.05	−6.25 ± 3.25	0.02*
Changes in the postoperative spherical equivalent refractive error	0.16 ± 2.42	−0.44 ± 1.24	0.39
Postoperative complications			
Macular pucker	0	1 (6.3)	0.23
Corneal epithelial disorder	7 (30.4)	1 (6.3)	0.07
Macular edema	1 (4.3)	0	0.4
Central serous chorioretinopathy	1 (4.3)	0	0.4
Cataract	1 (4.3)	0	0.4

WAVS, wide-angle viewing system; BCVA, best-corrected visual acuity.

*P < 0.05.
